# Success factors of health promotion: Evaluation by DEMATEL and M-DEMATEL methods — A case study in a non-profit organization

**DOI:** 10.1371/journal.pone.0260801

**Published:** 2021-12-07

**Authors:** Chi-Horng Liao, Silviu Bercea

**Affiliations:** 1 Tzu Chi University, Hualien, Taiwan; 2 University of Agricultural Sciences and Veterinary Medicine of Cluj-Napoca, Cluj-Napoca, Romania; University of Defence in Belgrade, SERBIA

## Abstract

Proper health knowledge and adequate motivation for health activities are key factors that influence an individual to adopt a healthy behavior. Health promotion positively influences progressive behaviors that seek to advance health potential, to continuously improve one’s lifestyle. There are many health promotion indications constantly encouraging people to eat healthier food. Based on the successful experience of a non-profit organization promoting a healthier vegetarian diet, this research identifies the operating factors that lead to the success of health promotion. The formulation and implementation of the health promotion strategy must be combined with the key success factors in order to accomplish the objectives. This study assessed seven factors, evaluated using the proposed method. The proposed Decision Making Trial and Evaluation Laboratory (DEMATEL) method constructs the cause and effect model of health promotion, and places forward suggestions and strategies for improvement based on the evaluation of the results. This research compared the original DEMATEL with a Modified DEMATEL (M-DEMATEL) to identify the success factors of health promotion. According to the results of both methods, “leadership”, “communication channel” and “budget” are the most important and influential factors when promoting healthy diets. The results have shown the connection and the difference between the two methods. The main purpose of this research is not to determine which method is the best method, instead, to derive the combined effect of both methods.

## Introduction

The change in disease patterns and many health problems are due to human life style and behavior. Medical models of disease treatment of the past have are not enough to meet the current health needs, as the concept of prevention needs a wider use in response to health problems. Every individual must take greater responsibility for their own health [1]. Solving a nation’s health problems is done primarily through long-term efforts to promote health and to prevent disease. Prevention is more important than treatment, and daily physical activity is the key to health care, preventing the occurrence of diseases and reducing unnecessary medical expenses, which is also an individual’s responsibility to the family and society.

Preventive medicine includes a variety of measures taken to prevent disease. Diseases and disabilities are influenced by environmental factors, genetic causes, disease vectors, and lifestyles, a dynamic process that occurs before an individual becomes aware of the impact. Preventive medicine has become particularly important as reflected by the global prevalence of chronic diseases and the deaths caused by these diseases. There are many ways to prevent disease. One of the examples is to encourage a vegetarian diet by providing health information [2]. Adults and children are advised to see a physician regularly, even if they feel healthy, to have a health check-up, to screen for diseases, to look for risk factors for diseases, to discuss health and balance lifestyle skills, to learn about the latest immunization and promotion altogether, and to maintain good interaction with medical professionals. Thus, the concept of health promotion is introduced to prevent disease and illness.

Experts and scholars have come up with various concepts for health promotion. Some scholars aim to practice a healthy lifestyle [3, 4], some aim at positive health [5, 6], and some see health promotion as a "process" for health [7], while others see it as the ultimate "result" [8]. Different definitions will influence the development of health promotion schemes and the choice of intervention strategies. Proper health knowledge and adequate health behavior motivation are key factors that influence whether an individual adopts a healthier behavior [9]. Many government agencies and scholars developed health education plans and health promotion interventions to promote health knowledge, health benefits of healthy behavior and the skills needed to educate people to conduct healthy behaviors, with the ultimate goal of making up their minds to initiate health promotion actions when people have sufficient knowledge and motivation [10, 11]. One of the benefits of health promotion is to expect the target audience to strengthen and enhance above mention messages into their own health knowledge, which in turn will affect people’s behavior and are beneficial to their own, as well as to society’s health [12].

This study uses Decision Making Trial and Evaluation Laboratory (DEMATEL) and Modified DEMATEL (M-DEMATEL) methods to construct the cause and effect model of health promotion, and places forward suggestions and strategies for improvement based on the evaluation results. The DEMATEL method had been widely applied to examine the relationships between various perceptions on complex subject [13]. It is hoped that, through the results and recommendations of the study, a suggestion can be provided for the health promotion practitioner, non-profit organization, healthcare agencies or government agencies to promote a health related action.

## Literature review

### Health promotion

The World Health Organization defined health promotion as the process by which people and communities can strengthen their control over health determinants [14]. This process requires the direct involvement of individuals and communities to complete the change, guided by political activity, to create a healthy environment. Health promotion refers to the combination of education and environmental support to encourage people to take healthy actions and lifestyle [15]. Hence, health promotion includes health education, policy and the environment, as healthy people engage in health-friendly activities in order to lead healthier lives [10]. The target audience of health promotion is usually healthy people that undertake healthy behaviors and activities. Health promotion also contributes to increasing activity levels of health and achieving individual, family, community, and social health [16]. Thus, health promotion is a sum of progressive behaviors that seek to advance health potential, to continuously improve one’s lifestyle. Health promotion helps people change their lifestyles by combining awareness, behavior change, environmental creation, as well as other factors [17]. In short, health promotion encourages people to improve their lives towards better health.

Health promoting behavior helps people go from a state of unhealthy lifestyle to a better state of health and well-being. Pender [16] points out that health promotion is an approach behavior for the development of healthy potential, including any activity oriented towards the level of personal, family, community and social wellness. This describes the scope of health promotion, covering individual, family, and social well-being. Shamansky and Clausen [18] also suggest that health promotion behavior includes the management of the body and emotions of the individual, with a greater emphasis on nutrition, exercise, hygiene habits, avoidance of risk factors, increased body immunity and other behaviors. Health Promotion encourages people to avoid lifestyles that are harmful to health, including smoking, alcohol consumption and substance abuse. Aside from the above-mentioned avoidance preventive behavior, van der Put [19] and Mulderij [20, 21] also comprise physical and fitness activities, family planning, mental health, education, community and other related behaviors relating to health promotion. Although health promotion-related behaviors do not necessarily mean that individuals have a healthy lifestyle, it is an indispensable factor in developing a healthier lifestyle.

There are many hidden issues in people’s health, especially chronic and oncological diseases. There are many ways to prevent illness; and one of the recommended behaviors is the vegetarian diet. Many health promotion materials constantly encourage people to take a vegetarian diet. Eating more fruits and vegetables can prevent colorectal cancer, high blood pressure, cardiovascular disease, as well as other health issues, which also means that vegetables are helpful for the health of the body [22]. The vegetarian diet is low in fat, cholesterol and calories, and rich in fiber, phytochemicals, vitamins and minerals and other nutrients, so it is easy to feel full, which helps to control weight, prevent obesity, control cardiovascular disease, reduce cancer and diabetes risk, promote body metabolism, and so on [2, 23]. Therefore, by promoting a vegetarian diet, it would help people realize the above-mentioned benefits. This research focuses on the health promotion of a vegetarian diet.

The formulation and implementation of the health promotion strategy must be combined with the key success factors in order to accomplish the objectives. The Key Success Factor is one of the methods of planning information system development [24]. This concepts used to explore the relationship between industrial attributes and corporate strategy, in which to combine the corporate specific ability with a response to the requirements of the environment in order to achieve the organizational goal [25]. The approach is to identify the key success factors that make the organization successful through analysis, and then to determine the necessities of the system and plan throughout these key factors [26]. Aside from organizational success factors, a number of studies identified the success factors in relation with health promotion, as further discussed.

The framework that we present in this study makes a worthwhile contribution as it defines critical factors that were not previously covered in literature. Furthermore, adhering to these factors will ensure a facile implementation of health promotion.

This research introduces one of the multi-criteria decision analysis methods–DEMATEL. This method serves to first simplify the multi-factor system structure and then screen out the main influence factors. Based on graph theory, DEMATEL supports the development of knowledge and experience; it allows for analysis of the logical correlations and direct-influence relationships between various factors in complex systems, thus revealing key factors [27]. The main objective is usually to establish a ranking of alternatives, where the best solution is in first place and the least important is in the last place [3].

Furthermore, this study provides an overview of the DEMATEL method with a detailed illustration of the steps that the authors find crucial in the DEMATEL, and which are often still not understood by researchers. Conducting the DEAMTEL is a time-consuming activity, and some steps are very challenging. Therefore, the authors introduce the readers to M-DEMATEL, a less complex method, appropriate for practitioners.

### Factors of health promotion

There were several previous studies that investigated a wide range of health promotion success factors as independent variable in evaluating the contribution to the effectiveness. Various health promotion success factors have been assessed separately. Of the more recent studies we can name: Puška et al. [28], Legrand et al. [29] and Maijala et al. [30] evaluated administration and management, while Cramm et al. [31] and Maijala et al. [30] researched the implementation of skills and resources. Retrum et al. [32], Juel et al. [33] and Rongen et al. [34] measured the benefits associated with participants. Mahmud et al. [35] and Kreps et al. [36] discussed communication efforts in various applications. Maijala et al. [30], Zahner et al. [37] and Kun et al. [38] examined the influence of budget. Cramm et al. [31], Kholifah et al. [39], Jones et al. [40], Darlington et al. [41] and Kennedy et al. [42] investigated the importance of self-efficiency. Chen and Lee [10], Stone et al. [43] and Aziato et al. [44] and recommended leadership as the most important factor of implementing health promotion. Based on the work of these researchers, this study selected seven of the most assessed factors to be evaluated using the proposed method: Budget, Communication channel, Benefits associated with participant, Administration and management, Leadership, Self-efficacy, Skills and Resources. The evaluations are as follows.

#### Budget (H1)

A budget is a detailed statement of the resources available to a program or activity and what it costs to implement the program [45]. A health promotion program may have a more complicated budget, with multiple funding streams, various expenses, and anticipated changes in both expenses and incomes at various program stages. Thus, it is important to have enough budget to operate any health promotion program.

#### Communication channel (H2)

The communication channel is a type of media that is used to transfer a message from one person or an organization to another. The chosen channel should be accessible to the health planner and credible to the participant [46]. In a non-profit organization specifically, communication channels are the way information flows in the organization within, and with other individuals and society. In health promotion, the communication campaigns, mass media and health-related product distribution have been used to reduce mortality and morbidity through behavioral change [31].

#### Benefits associated with participant (H3)

Health promotion is aimed to influence the social health behavior and the purpose is to improve the health of the people and society. H3 is a strategic approach to focus on generating and delivering valuable, relevant, and consistent health promotion content to attract and maintain the target participant. Thus, the health promotion practitioner should provide a useful framework for systematically understanding the benefits of the health products to the participant [47].

#### Administration and management (H4)

A quality administration and management system of health promotion is required to lead a successful program. Administrative support must be furnished to new program operations and modified accordingly; the period ahead will involve the redirection of administration and management resources as directed to the program needs. This is to say that administration and management must be prepared to implement program decisions with policies and procedures responsive to the program needs with evolved management support required to assure the successful achievement of health promotion program goals [48].

#### Leadership (H5)

Leadership was the factor most often empirically related to health promotion effects achieved through cooperative work [43]. Primary health care leaders’ appreciation towards health promotion is important. Charisma and personality traits of the leader can make the followers sincerely admire and respect the person [49], and the leader’s good abilities at caring, motivational skills and the attitude of leading by example will set the leader as the role model of the followers and motivate compliance.

#### Self-efficacy (H6)

Self-efficacy is the perception of whether to engage in a healthy behavioral shift, how much effort it takes, and how long it lasts in the face of difficulties and failures. Self-efficacy affects the degree at which people set health goals. Self-efficacy is measured through some studies of health practice to assess their potential role in health behavioral change [50].

#### Skills and resources (H7)

The organization should make sure that there are sufficient skills and resources for health promotion practices. The ability to work effectively among the members and community is also an important factor leading to the success of a health promotion activity. The financial and human resources needed and promotion skills required should be identified before the health promotion program is started [51].

## Materials and methods

**Fig 1:**
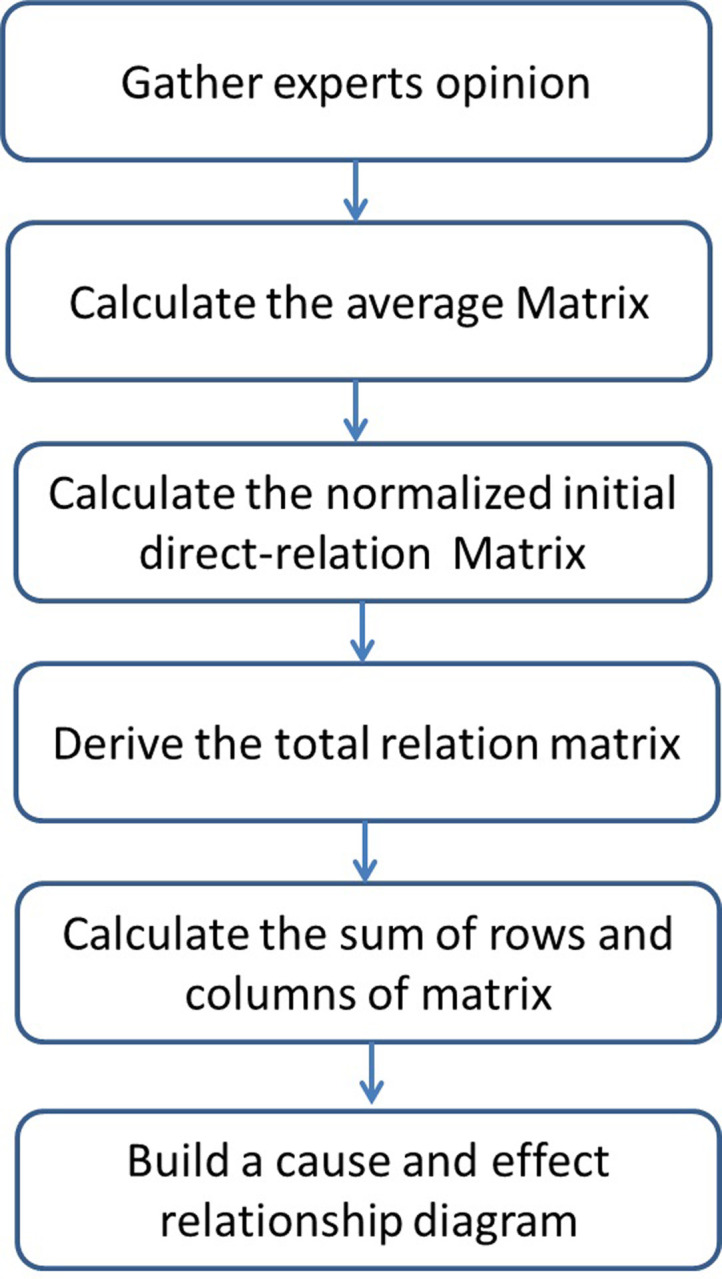
The process of the DEMATEL method.

The DEMATEL method was developed by the Banelle Memorial Institute of Geneva between 1972 and 1976 for the Science and Human Affairs Program to solve complex problems [52]. The DEMATEL method can improve the understanding of specific problems, entangle cluster relation, and, through the use of hierarchical structure, provide feasible identical solutions [13]. DEMATEL typically illustrates the interrelationship among key factors in order to obtain the core guidelines that effectively represent these factors [53]. DEMATEL has been successfully applied in many fields. According to the study of Koca and Yıldırım [54], the uses of the DEMATEL method were concentrated in fields such as computer science and artificial intelligence, environmental science, operations research and management science, management, green sustainable technologies, electrical electronics engineering, and industrial engineering. In relationship to hospital management, some scholars used DEMATEL to investigate or evaluate the hospital service quality [55], hospital accreditation standards [56], hospitals outpatient service [57], hospital supply chain performance [58], medical service system [59] and medical device development [60]. In connection with healthcare, some scholars applied DEMATEL to analyze and explore healthcare industry [61, 62], lean healthcare management [63, 64], healthcare waste treatment technologies [65], and healthcare service quality [66]. Furthermore, the DEMATEL method intends to find direct and indirect relationships, and to gauge the strength of influence between different factors in a complex environment [67]. Thus, DEMATEL is considered a suitable methodology for this research (Fig 1). In addition, this research intends to compare the original DEMATEL with M-DEMATEL to identify the success factors of health promotion. The procedures of these two comparison methods can be summarized as follows [52, 68, 69].

### DEMATEL method

Step 1: Collecting data from participants and deriving the initial matrix of each participant
  Assume that there are *n* factors and *p* participants involved in the research. The initial matrices of *Z*^(1)^, *Z*^(2)^, …, *Z*^(*p*)^ is the measure score from *p* experts. Let (i, j) element of initial matrix *Z*^(*k*)^ is denoted by $z_{ij}^{\left(k\right)}$, and $z_{ij}^{\left(k\right)}$ represents the pair of degree of effect on i factor effects j factor of expert *k*. These pairwise comparisons given an integer score of 0, 1, 2, 3 or 4, representing ‘no influence’, ‘low influence’, ‘medium influence’, ‘high influence’, and ‘very high influence’, correspondingly. The elements for *i* = *j* are set to 0.
  Average Step (of DEMATEL Method): Construct initial direct-relation matrix *Z*
  An initial direct-relation matrix *Z* with individual element *z*_*ij*_ can be expressed in Eq (1):
$$Z={\left[z_{ij}\right]}_{n\times n}\;\text{where}\;z_{ij}=\frac{1}{p}\;\sum_{s=1}^{p}z_{ij}^{\left(s\right)}$$ (1)
Step 2: Compute the normalized direct-relation matrix *X*
  The normalized direct-relation matrix *X* can be derived from Eq (2), where the matrix diagonal is coded 0 and the sum of each row and column does not exceed 1.
$$X={\left[\frac{z_{ij}}{u}\right]}_{n\times n},\;where\;u=max\left\{\underset{i}{max}\sum_{j=1}^{n}z_{ij},\;\underset{j}{max}\sum_{i=1}^{n}z_{ij}\right\}$$ (2)
Step 3: Attaining the total-relation matrix *T*
  The total-relation matrix *T* can be obtained by using Eq (3). This illustrates the final structure of elements after infinite series of direct effects.
$$T={\left[t_{ij}\right]}_{n\times n}=\sum_{s=1}^{\infty }X^{s}=X{\left(I-X\right)}^{-1},\;\text{where}\;I\;\text{is}\;\text{an}\;\text{identity}\;\text{matrix}$$ (3)
Step 4: Identify the cause and effect groups
  Base from the result of total-relation matrix *T*, the rows sum and columns sum are separately indicated as vector *R* and *C* through Eqs (4) and (5). The *r*_*i*_ summarize effects given by factor *i* to other factors, whereas *c*_*j*_ summarize the total effect received by factor *j* from other factors. Accordingly, (*r*_*i*_ + *c*_*j*_) indicates the level of importance that factor *i* plays in the entire framework. Besides, (*r*_*i*_ − *c*_*j*_) indicates the degree of net effect that factor *i* contributes to the decision framework. If (*r*_*i*_ − *c*_*j*_) is positive, the factor *i* is a net cause, implying that others are influenced by factor *i*; if (*r*_*i*_ − *c*_*j*_) is negative, the factor *i* is a net effect, implying that others influence the factor *i*.
$$R={\left[r_{i}\right]}_{n\times 1}={\left[\sum_{j=1}^{n}t_{ij}\right]}_{n\times 1}$$ (4)
$$C={\left({\left[c_{j}\right]}_{1\times n}\right)}^{T}={\left({\left[\sum_{i=1}^{n}t_{ij}\right]}_{1\times n}\right)}^{T}$$ (5)
Step 5: Designing the casual and relation map
  Establish and analyze the structural model. The casual and relation map can be derived by mapping the numerical values of (*r*_*i*_ + *c*_*j*_, *r*_*i*_ − *c*_*j*_), where (*r*_*i*_ + *c*_*j*_) is the horizontal axis and (*r*_*i*_ − *c*_*j*_) is the vertical axis.

### M-DEMATEL method

This research extends DEMATEL to M-DEMATEL to identify the success factors of health promotion. By adjusting the structure of computation, it is possible that the sequence of the success factors and causal effect influence may differ. M-DEMATEL can be an alternative for a decision making process. The procedures of M-DEMATEL are as follows.

Step 1: Collecting data from experts and deriving the initial matrix of each expert
  Suppose that there are *n* factors and *p* experts engaged in the research. The initial matrices of *Z*^(1)^, *Z*^(2)^, …, *Z*^(*p*)^ is the measure score from *p* experts. Let (*i*, *j*) component of initial matrix *Z*^(*k*)^ is expressed by $z_{ij}^{\left(k\right)}$, and $z_{ij}^{\left(k\right)}$ signifies the pair of degree of effect on *i* factor effects *j* factor of expert *k*. These pairwise comparisons given an integer score of 0, 1, 2, 3 or 4 representing the degrees of influence. The elements for *i* = *j* are set to 0.
Step 2: Compose the normalized direct-matrix *X*^(*k*)^ of each expert for *k* = 1,2,…,*p*
  After constructing the initial direct-relation matrix of each expert, this research design not to compute the average value of all the experts, instead, go direct to compute the normalized direct-matrix of each expert. The average step would only be process after the calculation of each expert’s total-relation matrix. The normalized direct-relation matrix *X*^(*k*)^ of each expert can be obtained by Eq (6), where the sum of each row and column does not exceed 1 and the matrix diagonal is coded 0.
$${\text{X}}^{\left(\text{k}\right)}={\left[\frac{{\text{z}}_{\text{ij}}^{\left(\text{k}\right)}}{{\text{u}}^{\left(\text{k}\right)}}\right]}_{\text{n}\times \text{n}},\;{\text{where}\;\text{u}}^{\left(\text{k}\right)}=\text{max}\left\{\underset{\text{i}}{\max}\sum_{\text{j}=1}^{\text{n}}{\text{z}}_{\text{ij}}^{\left(\text{k}\right)},\;\underset{\text{j}}{\max}\sum_{\text{i}=1}^{\text{n}}{\text{z}}_{\text{ij}}^{\left(\text{k}\right)}\right\}$$ (6)
Step 3: Derive the total-relation matrix *T*^(*k*)^ of each expert for *k* = 1,2,…,*p*
  The total-relation matrix *T*^(*k*)^ of each expert can be obtained by Eq (7), in which *I* is signified as the identity matrix.
$$T^{\left(k\right)}={\left[t_{ij}^{\left(k\right)}\right]}_{n\times n}=\sum_{s=1}^{\infty }{\left(X^{\left(k\right)}\right)}^{s}=X^{\left(k\right)}{\left(I-X^{\left(k\right)}\right)}^{-1}$$ (7)
  Average Step (of *M-DEMATEL Method*): Compute the modify total-relation matrix *T*′. The modify total-relation matrix *T*′ is derived using Eq (8).
$$T\prime ={\left[t_{ij}^{\prime }\right]}_{n\times n}\;\text{where}\;t_{ij}^{\prime }=\frac{1}{p}\;\sum_{s=1}^{p}t_{ij}^{\left(s\right)}$$ (8)
Step 4: Calculation of cause and effect groups
  The column sum and the row sum are individually indicated as vectors *R′* and *C′* within the modify total-relation matrix *T′*, which would be obtained from Eqs (9) and (10). The value *r*_*i*_*′* summarized the influences allocated by factor *i* to other factors, whereas *c*_*j*_*′* summarized the total effect received by factor *j* from other factors. The vector (*r*_*i*_*′* + *c*_*j*_*′*) is called a prominence whereas the vector (*r*_*i*_*′* − *c*_*j*_*′*) is called a relation. If the value of (*r*_*i*_*′* − *c*_*j*_*′*) is positive, the factor is a net causer which belongs to the cause group. However, if the value of (*r*_*i*_*′* − *c*_*j*_*′*) is negative, the factor is a net receiver which belongs to the effect group.
$$R^{\prime }={\left[r_{i}^{\prime }\right]}_{n\times 1}={\left[\sum_{j=1}^{n}t_{ij}^{\prime }\right]}_{n\times 1}$$ (9)
$$C^{\prime }={\left({\left[c_{j}^{\prime }\right]}_{1\times n}\right)}^{T}={\left({\left[\sum_{i=1}^{n}t_{ij}^{\prime }\right]}_{1\times n}\right)}^{T}$$ (10)
Step 5: Designing the causal relationship map
  Establish and analyze the structural model. The casual and relation map can be derived by mapping the numerical values of (*r*_*i*_ + *c*_*j*_, *r*_*i*_ − *c*_*j*_), where (*r*_*i*_ + *c*_*j*_) is the horizontal axis and (*r*_*i*_ − *c*_*j*_) is the vertical axis.

### Data collection

When applying the DEMATEL method, the underlying assumption is that the opinions from all of the respondents should be taken into consideration [55]. Theoretically, the suggested maximum number of participants in the decision-making process is 20, in order to avoid inconsistency [70]. The questionnaires were primarily administered to a group of experts, who provided their personal opinions regarding the health promotion of a vegetarian diet. The individual responses from each expert were collected separately and further aggregated, in order to be used as an input to DEMATEL method.

The respondents were volunteers and members of the Tzu Chi foundation, a foundation dedicated to medicine, education, humanity, environment protection, and international relief, established in 1966 by Dharma Master Cheng Yen [71]. Of the 20 respondents contacted, a number of 12 agreed to participate in the study, each having at least 15 years of experience in promoting healthy lifestyles. None of the participants were excluded or withdrew from the study.

## Results

Based on the review of literature, seven success factors of health promotion were identified. These factors are Budget (H1); Communication channel (H2); Benefits associated with participant (H3); Administration and management (H4); Leadership (H5); Self-efficiency (H6); Skills and Resources (H7). The aim of applying the DEMATEL method is not only to determine the important factors of health promotion, but at the same time to measure the causal relationships among factors. Furthermore, this research also attempted to determine the differences between the outcome of DEMATEL and M-DEMATEL methods. As shown in Table 1, the average step of DEMATEL was adjusted in the later part as indicated in M-DEMATEL.

**Table 1:** The procedures of DEMATEL and M-DEMATEL.

Steps	DEMATEL	M-DEMATEL
Step 1: Construct initial matrix	Input *Z*^(*k*)^	Input *Z*^(*k*)^
Average step (for DEMATEL only) Compute initial direct-relation matrix	*Z* = avg(*Z*^(*k*)^)	-
Step 2: Compose normalized direct-matrix	*X = Z / u*	*X*^(*k*)^ = *Z*^(*k*)^/*u*^(*k*)^
Step 3: Attaining total-relation matrix	*T* = *X*(*I* − *X*)^-1^	*T*^(*k*)^ = *X*^(*k*)^(*I* − *X*^(*k*)^)^-1^
Average step (for M-DEMATEL only) Compute modify total-relation matrix	-	T’ = avg(*T*^(*k*)^)
Step 4: Calculation of cause and effect groups	*R* and *C*	*R′* and *C′*
Step 5: Design causal relationship map	(*r*_*i*_ + *c*_*j*_, *r*_*i*_ − *c*_*j*_)	(*r*_*i*_*′* + *c*_*j*_*′*, *r*_*i*_*′* − *c*_*j*_*′*)

### DEMATEL method

The opinions of each expert were collected and averaged in order to formulate the initial direct-relation matrix as shown in Table 2. The normalization of the direct-matrix was calculated by dividing the sum of each intersection value to the maximum value of the columns sum and the row sum of the initial direct-relation matrix which is 15.5. The outcome of the normalized direct-matrix *X* is shown in Table 3. The total-relation matrix is obtained from the normalized direct-matrix. The result of the total-relation matrix is presented in Table 4. In the tables of the DEMATEL method, the symbol of H1G represents the H1: Budget; H2G represents the H2: Communication channel and so on with the other 5 factors.

**Table 2:** The DEMATEL initial direct-relation matrix.

*Z*	H1G	H2G	H3G	H4G	H5G	H6G	H7G
H1G	0.0000	2.5833	2.5000	2.5833	2.5000	2.5000	2.5833
H2G	2.2500	0.0000	2.5000	2.5000	2.7500	2.6667	2.7500
H3G	2.5000	2.2500	0.0000	2.5833	2.2500	2.6667	2.8333
H4G	2.0000	2.4167	2.3333	0.0000	2.6667	2.4167	2.3333
H5G	2.5000	2.5833	2.5833	2.5833	0.0000	2.5000	2.5000
H6G	2.4167	2.4167	2.3333	2.3333	2.2500	0.0000	2.5000
H7G	2.5000	2.3333	2.5000	2.0833	2.3333	2.3333	0.0000

Budget (H1); Communication channel (H2); Benefits associated with participant (H3); Administration and management (H4); Leadership (H5); Self-efficiency (H6); Skills and Resources (H7).

**Table 3:** The normalized direct-matrix X.

*X*	H1G	H2G	H3G	H4G	H5G	H6G	H7G
H1G	0.0000	0.1667	0.1613	0.1667	0.1613	0.1613	0.1667
H2G	0.1452	0.0000	0.1613	0.1613	0.1774	0.1720	0.1774
H3G	0.1613	0.1452	0.0000	0.1667	0.1452	0.1720	0.1828
H4G	0.1290	0.1559	0.1505	0.0000	0.1720	0.1559	0.1505
H5G	0.1613	0.1667	0.1667	0.1667	0.0000	0.1613	0.1613
H6G	0.1559	0.1559	0.1505	0.1505	0.1452	0.0000	0.1613
H7G	0.1613	0.1505	0.1613	0.1344	0.1505	0.1505	0.0000

Budget (H1); Communication channel (H2); Benefits associated with participant (H3); Administration and management (H4); Leadership (H5); Self-efficiency (H6); Skills and Resources (H7).

**Table 4:** The total-relation matrix.

*T*	H1G	H2G	H3G	H4G	H5G	H6G	H7G
H1G	2.7798	2.9902	3.0156	3.0020	3.0135	3.0704	3.1449
H2G	2.9314	2.8723	3.0409	3.0230	3.0505	3.1041	3.1792
H3G	2.8874	2.9418	2.8440	2.9693	2.9686	3.0450	3.1228
H4G	2.7235	2.8069	2.8305	2.6832	2.8445	2.8860	2.9480
H5G	2.9194	2.9909	3.0203	3.0028	2.8753	3.0712	3.1417
H6G	2.7563	2.8198	2.8435	2.8269	2.8375	2.7644	2.9698
H7G	2.7371	2.7917	2.8273	2.7908	2.8169	2.8706	2.8058

Budget (H1); Communication channel (H2); Benefits associated with participant (H3); Administration and management (H4); Leadership (H5); Self-efficiency (H6); Skills and Resources (H7).

The direct influences of health promotion factors were indicated in Table 5. First, the importance of the degree of each factor (*r*_*i*_ + *c*_*j*_) was calculated. The (*r*_*i*_ + *c*_*j*_) value is considered important if the (*r*_*i*_ + *c*_*j*_) result is greater than the average value (40.9141). Based on the data, the four important factors are H2: Communication channel; H3: Benefits associated with participant; H5: Leadership; H7: Skills and Resources. The ranking according to the importance of health promotion is as follows: H5 > H2 > H3 > H7 > H1 > H6 > H4.

**Table 5:** The direct and indirect influence of factors.

	*R*	*C*	*r*_*i*_ + *c*_*j*_	*r*_*i*_ − *c*_*j*_
H1G	21.0164	19.7349	40.7513	1.2815
H2G	21.2016	20.2135	41.4151*	0.9881
H3G	20.7788	20.4221	41.2009*	0.3568
H4G	19.7225	20.2979	40.0205	-0.5754
H5G	21.0217	20.4069	41.4286*	0.6148
H6G	19.8180	20.8117	40.6297	-0.9937
H7G	19.6402	21.3122	40.9524*	-1.6721

Budget (H1); Communication channel (H2); Benefits associated with participant (H3); Administration and management (H4); Leadership (H5); Self-efficiency (H6); Skills and Resources (H7).

**Fig 2:**
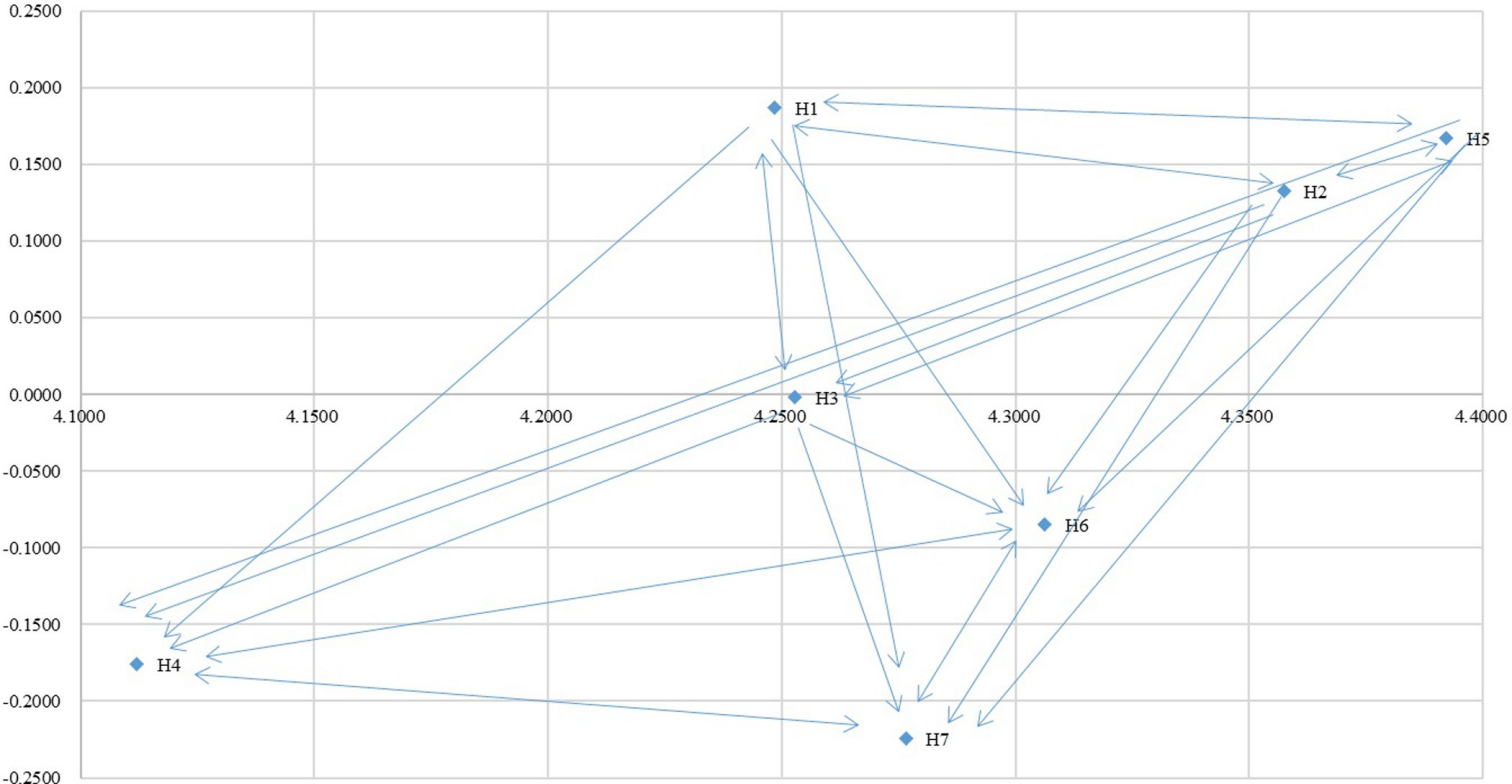
Network relationship map of influences among factors of DEMATEL. Budget (H1); Communication channel (H2); Benefits associated with participant (H3); Administration and management (H4); Leadership (H5); Self-efficiency (H6); Skills and Resources (H7).

Further, the (*r*_*i*_ − *c*_*j*_) value for each factor is calculated, to examine the influence of each factor, as it has either a positive (as an influencer) or a negative (as a recipient) impact. Out of the seven factors, four factors (i.e., H1: Budget; H2: Communication channel; H3: Benefits associated with participant; H5: Leadership) were evaluated as influencer. And, the remaining three factors (i.e., H4: Administration and management; H6: Self-efficiency; H7: Skills and Resources) were evaluated as recipient (see Table 5). The cause and effect diagram constructed based upon the prominence (*r*_*i*_ − *c*_*j*_) value and relation value (*r*_*i*_ − *c*_*j*_) is shown in Fig 2.

### M-DEMATEL method

As discussed, and presented in Table 1, the average step would only be processed after calculation of the total-relation matrix of each expert in M-DEMATEL method. After the constructed initial direct-relation matrix of each expert, the normalized direct-matrix of each expert was composed. The maximum value of the columns sum and the row sum of initial direct-relation matrix shall be determined beforehand. The maximum values from initial direct-relation matrix from expert 1 to expert 12 are 22.0, 22.0, 22.0, 22.0, 23.0, 18.0, 24.0, 20.0, 21.0, 23.0, 22.0, and 24.0. The normalized direct-matrix of the 12 experts is shown in Supporting information A section, SA1-SA12 Tables in S1 File. In the tables of M-DEMATEL method, the symbol of H1F represents the H1: Budget; H2F represents the H2: Communication channel and so on with the other 5 factors.

After the normalized initial direct-relation matrix, the next procedure is to attain the total-relation matrix of each expert. The results of the total-relation matrix of each expert are shown in Supporting information B section, SB1-SB12 Tables in S1 File. Based on the result of each expert’s total-relation matrix, the next step is to compute the modified total-relations matrix of the 12 experts. For example: T′(1,1) = (T^(1)^(1,1) + T^(2)^(1,1) + …+ T^(12)^(1,1)) / 12 = (0.3912 + 0.0882 + 0.0965 + 0.3356 + 0.3444 + 0.0965 + 0.3617 + 0.1442 + 0.1108 + 0.2954 + 0.1559 + 0.1315) / 12 = 0.2126. The result of the modified total-relation matrix is shown in Table 6.

**Table 6:** Modify total-relation matrix.

*T′*	H1F	H2F	H3F	H4F	H5F	H6F	H7F
H1F	0.2126	0.3270	0.3319	0.3356	0.3265	0.3337	0.3505
H2F	0.3093	0.2260	0.3334	0.3366	0.3382	0.3443	0.3572
H3F	0.3088	0.3073	0.2096	0.3222	0.3054	0.3332	0.3390
H4F	0.2747	0.2968	0.2919	0.1958	0.3036	0.3018	0.3036
H5F	0.3232	0.3392	0.3386	0.3464	0.2324	0.3469	0.3528
H6F	0.3052	0.3129	0.3136	0.3143	0.3058	0.2249	0.3342
H7F	0.2970	0.3032	0.3084	0.2929	0.3006	0.3107	0.2132

Budget (H1); Communication channel (H2); Benefits associated with participant (H3); Administration and management (H4); Leadership (H5); Self-efficiency (H6); Skills and Resources (H7).

The next step is to determine the *R′* and *C′* values based on the modified total-relation matrix, to identify the cause and effect groups. The column sum and the row sum were added and shown in Table 7. For example:*r*_1_*′* = 0.2126 + 0.3270 + 0.3319 + 0.3356 + 0.3265 + 0.3337 + 0.3617 + 0.3505 = 2.2177.

**Table 7:** The value of *R′* & *C′* and identify cause and effect group.

	*R′*	*C′*	*r*_*i*_*′* + *c*_*j*_*′*	*r*_*i*_*′* − *c*_*j*_*′*
H1F	2.2177	2.0307	4.2485	0.1870
H2F	2.2450	2.1124	4.3574*	0.1326
H3F	2.1255	2.1273	4.2528	-0.0019
H4F	1.9682	2.1439	4.1121	-0.1758
H5F	2.2796	2.1125	4.3921*	0.1671
H6F	2.1108	2.1954	4.3062*	-0.0846
H7F	2.0261	2.2505	4.2766	-0.2244

**Fig 3:**
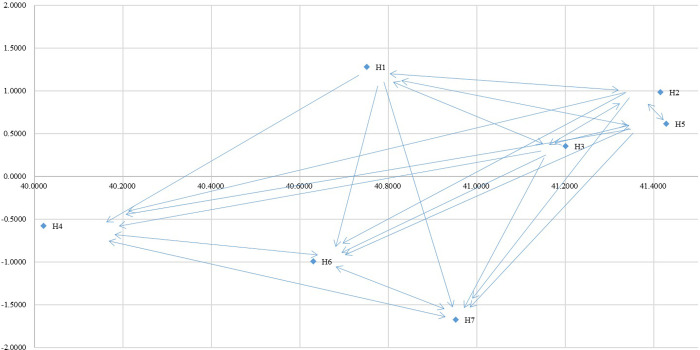
Network relationship map of influences among factors of M-DEMATEL. Budget (H1); Communication channel (H2); Benefits associated with participant (H3); Administration and management (H4); Leadership (H5); Self-efficiency (H6); Skills and Resources (H7).

Following M-DEMATEL, the values of (*r*_*i*_*′* + *c*_*j*_*′*) were calculated which represents the degree of importance of each factor. The data indicated in Table 7 presented the three most important factors. These factors are H2: Communication channel, H5: Leadership and H6: Self-efficacy, which means that the values of these three factors are greater than the average value of 4.2779. The overall ranking of importance of the health promotion factors is as follow: H5 > H2 > H6 > H7 > H3 > H1 > H4. Furthermore, the (*r*_*i*_*′* − *c*_*j*_*′*) values segregate the factors into cause and effect group depending upon the positive and negative values of each factor. Among all the factors, H1: Budget, H2: Communication channel and H5: Leadership are positive which signify as the cause factors. The remaining four factors of the H3: Benefits associated with participant, H4: Administration and management, H6: Self-efficacy and H7: Skills and Resources are negative value which consider as the effect factors. The result of (*r*_*i*_*′* + *c*_*j*_*′*, *r*_*i*_*′* + *c*_*j*_*′*) are shown in Table 7. The cause and effect diagram shows the causal relationships among factors, and demonstrates how each factor directly influences the other factors of health promotion (see Fig 3).

## Discussion

### Evaluation for DEMATEL method

The results of the DEMATEL method indicate that “Budget” has the highest (*r*_*i*_*′* − *c*_*j*_*′*) value (1.2815), but, with below average (*r*_*i*_*′* + *c*_*j*_*′*) value (40.7516). This implies that “Budget” is the most influential factor among all. Therefore, it concludes that without sufficient funds, a health promotions campaign may not be functional in practice.

The results also showed that the (*r*_*i*_*′* + *c*_*j*_*′*) value of “Communicational Channel” (41.4151), “Leadership” (41.4286) and Benefits associated with participant (41.2009) are the important determinants. Also, the (*r*_*i*_*′* − *c*_*j*_*′*) value of “Communicational Channel” (0.9811), “Benefits associated with participant” (0.3568) and “Leadership” (0.6148) are positive values. This signifies that in order to promote health, raise awareness and induces changes in behavior, the non-profit organizations need to have a strong leadership to motivate the health promotion campaign, an effective communication channel to promote the healthy indications and an efficient strategy to maximize the benefit associated with target audiences.

“Skills and Resources” attains the (*r*_*i*_*′* − *c*_*j*_*′*) value of -1.6721 which indicates that this factor receives strong impact from cause group. However, it is the fourth important factor with (*r*_*i*_*′* + *c*_*j*_*′*) value of 40.9524. This result suggests that inducing the skills and resources of the volunteers are also important for promoting healthy indications.

### Evaluation for M-DEMATEL method

In case of M-DEMATEL, “Budget” also has the highest (*r*_*i*_*′* − *c*_*j*_*′*) value (0.1870) and below average (*r*_*i*_*′* + *c*_*j*_*′*) value (4.2485). This indicates that “Budget” has the maximal influence on other factors. Furthermore, the (*r*_*i*_*′* + *c*_*j*_*′*) value of “Communicational Channel” (4.3574), and “Leadership” (4.3574) are the following two most important factors. The (*r*_*i*_*′* − *c*_*j*_*′*) value of “Communicational Channel” (0.1326) and “Leadership” (0.1671) are also positive values. These two factors are important and influential. Lastly, “Self-efficiency” gets the (*r*_*i*_*′* − *c*_*j*_*′*) value of -0.0846 with the (*r*_*i*_*′* + *c*_*j*_*′*) value of 4.3062. This means that in order to have an effective health promotion campaign, the participant should have strong self-efficacy to implement the change of behavior.

### Comparisons of methods

According to the result of DEMATEL and M-DEMATEL methods, “Communication Channel” and “Leadership” are both important and belong to the influential factors. “Budget” is the most influential factor. “Administration and management” is of lower importance and belonging to the effect factor. Aside from these similarities, the following are the differences between the two methods.

First of all, the ranking of (*r*_*i*_*′* + *c*_*j*_*′*) values differs. The ranking of DEMATEL is H5 > H2 > H3 > H7 > H1 > H6 > H4. And, the ranking of M-DEMATEL is H5 > H2 > H6 > H7 > H3 > H1 > H4. The rankings are different after H2 in the comparison by these two methods. Furthermore, there are four factors that the (*r*_*i*_*′* + *c*_*j*_*′*) values are above average in DEMATEL. Then, there are only three factors that the (*r*_*i*_*′* + *c*_*j*_*′*) values are above average in M-DEMATEL. Secondly, the (*r*_*i*_*′* + *c*_*j*_*′*) value of “Skills and Resources” is above average in DEMATEL, but, below average in M-DEMATEL. However, the (*r*_*i*_*′* + *c*_*j*_*′*) value of “Self-efficiency” is below average in DEMATEL, but, above average in M-DEMATEL. Thirdly, there are four positive (*r*_*i*_*′* − *c*_*j*_*′*) values in DEMATEL. However, there are only three positive (*r*_*i*_*′* − *c*_*j*_*′*) values in M-DEMATEL. Lastly, “Benefits associated with participant” are both important and influential in DEMATEL. However, the (*r*_*i*_*′* + *c*_*j*_*′*) value of “Benefits associated with participant” is below average and the (*r*_*i*_*′* − *c*_*j*_*′*) value is negative in M-DEMATEL.

## Conclusions, limitations and recommendations

### Conclusions

This is the first research that applied DEMATEL and M-DEMATEL in the field of health promotion, as well as the first study that compared the DEMATEL method to the M-DEMATEL method. According to the result of both methods, “Leadership” and “Communication channel” are the most two important factors and also the influential factors when promoting healthy vegetarian diet, while “Budget” is the most influential factors among all.

“Leadership” is the ability to make subordinates obey voluntarily, and the key word is voluntary [72]. In a non-profit organization, the charisma, kindness and compassion of the leader drives the behavior of the volunteers and members. Therefore, leadership is authoritative when followers are willing to obey because they believe the leadeR′s directions represent followers’ self-interest and also the mission of the organization. Leadership was identified as the most frequently associated factor with health promotion [43].

In case of the “Communication channel”, health promotion may benefit from use of mass media to promote positive health behaviors [73]. In Taiwan, several non-profit organizations run a television station and broadcasting network for the communication of humanity, environmental protection and health promotion in order to persuade healthy behaviors. Thus, the result implied that health promotion campaigns that combine mass media and communication channels with distribution of free or reduced-price health-related products are effective in improving healthy behaviors [46].

It is believed that “Budget” is one of the important factors to operate activity or campaign for any organization. And, budget requirements depend on program focus, available resources, and incentives incorporated into the program and the specific health promotion activity. Most of the non-profit organizations depend largely on the donation of the public. Budget is important and crucial for non-profit organizations because it can be a way to achieve organizational sustainability and provide resources for campaigns and activities [74].

### Limitations and future recommendations

Aside from the above-mentioned factors, this study cannot conclude that the other factors are not important. The conclusion is based on the survey result of the non-profit organization in the field of health promotion. The result may vary if future researchers study these factors in other fields.

As per the results presented in this research, some improvement areas have been identified. As mentioned earlier, this is the first research that applied DEMATEL and M-DEMATEL in the field of health promotion. This is also the first study that compared the DEMATEL method and the M-DEMATEL method. The results have shown the connection and the difference between the two methods. Although, this cannot conclude or suggest which method derives better results, nevertheless, the difference should be noted. The result can imply that there are differences between the methods in the case of health promotion. Furthermore, the main purpose of this research is not to determine which method is the best method, instead, to derive the combined effect of both methods. Therefore, this study recommends that researchers apply the M-DEMATEL method in other fields, and to compare or combine the outcome with the DEMATEL method in future research. It is also suggested that future research should combine these methods in order to find the most feasible key success factors for decision making analysis.

In addition, from the perspective of applications, this study also has several implications for further research. First, the literature review shows that a series of modified DEMATEL approaches have been developed, but no or few studies have been done to compare the methods in the same or different groups. So, one recommendation for future research is for the evaluation and comparison of the advantages and limitation of different DEMATEL methods in order to help practitioners select the suitable one for the difficulty they face. Second, to analyze the complicated interrelations between factors accurately, many computations are involved in the extended DEMATEL models, which limit their applications. Thus, a software tool should be developed in the future to facilitate the implementation of the DEMATEL and M-DEMATEL methods. Finally, future research could apply the DEMATEL methodology and its variants to other situations and broader fields, not considered in the previous studies.

The top ranking have a more significant impact on the similarity than those further away, which is right in the decision-making domain [3]. Therefore, it is also suggested that future research calculate Spearman’s correlation coefficient or WS similarity coefficient to compare the reference ranking and the tested rankings.

## Supporting information

S1 File The normalized direct-matrix of the 12 experts is shown in SA1-SA12 Tables in S1 File. The results of the total-relation matrix of each expert are shown in SB1-SB12 Tables in S1 File. Based on the review of literature, the seven success factors of health promotion were identified. These factors are Budget (H1); Communication channel (H2); Benefits associated with participant (H3); Administration and management (H4); Leadership (H5); Self-efficiency (H6); Skills and Resources (H7). In the tables of M-DEMATEL method, the symbol of H1F represents the H1: Budget; H2F represents the H2: Communication channel and so on with the other 5 factors.
(DOCX)
