# Supplementary material for: Success factors of health promotion: Evaluation by DEMATEL and M-DEMATEL methods — A case study in a non-profit organization
Source: PLoS One. 2021 Dec 7;16(12):e0260801. doi: 10.1371/journal.pone.0260801 (PMC8651107; doi:10.1371/journal.pone.0260801)
Supplement: S1 File — The results of the total-relation matrix of each expert are shown in SB1-SB12 Tables in S1 File. Based on the review of literature, the seven success factors of health promotion were identified. These factors are Budget (H1); Communication channel (H2); Benefits associated with participant (H3); Administration and management (H4); Leadership (H5); Self-efficiency (H6); Skills and Resources (H7). In the tables of M-DEMATEL method, the symbol of H1F represents the H1: Budget; H2F represents the H2: Communication channel and so on with the other 5 factors. (DOCX) [file pone.0260801.s001.docx]

**Supporting information**

The normalized direct-matrix of the 12 experts is shown in Table SA1 to Table SA12, presented in **Supporting information section A**. The results of the total-relation matrix of each expert are shown in Table SB1 to Table SB12, presented in **Supporting information section B**. Based on the review of literature, the seven success factors of health promotion were identified. These factors are Budget (H1); Communication channel (H2); Benefits associated with participant (H3); Administration and management (H4); Leadership (H5); Self-efficiency (H6); Skills and Resources (H7). In the tables of M-DEMATEL method, the symbol of H1F represents the H1: Budget; H2F represents the H2: Communication channel and so on with the other 5 factors.

**Supporting information section A**

Table SA1. The normalized direct-matrix of expert 1

| $\boldsymbol{X}$ | $\mathbf{H1F}$ | $\mathbf{H2F}$ | $\mathbf{H3F}$ | $\mathbf{H4F}$ | $\mathbf{H5F}$ | $\mathbf{H6F}$ | $\mathbf{H7F}$ |
| --- | --- | --- | --- | --- | --- | --- | --- |
| $H1F$ | 0.0000 | 0.1818 | 0.1364 | 0.1818 | 0.1364 | 0.1818 | 0.1818 |
| $H2F$ | 0.1364 | 0.0000 | 0.1364 | 0.1818 | 0.1818 | 0.1364 | 0.1818 |
| $H3F$ | 0.0909 | 0.1818 | 0.0000 | 0.1364 | 0.1364 | 0.1818 | 0.1364 |
| $H4F$ | 0.0455 | 0.0909 | 0.0909 | 0.0000 | 0.0909 | 0.0909 | 0.0909 |
| $H5F$ | 0.1818 | 0.1364 | 0.1364 | 0.1818 | 0.0000 | 0.1818 | 0.1818 |
| $H6F$ | 0.1364 | 0.1364 | 0.1364 | 0.1364 | 0.1364 | 0.0000 | 0.1364 |
| $H7F$ | 0.0455 | 0.0909 | 0.0455 | 0.0455 | 0.0455 | 0.0909 | 0.0000 |

Table SA2. The normalized direct-matrix of expert 2

| $\boldsymbol{X}$ | $\mathbf{H1F}$ | $\mathbf{H2F}$ | $\mathbf{H3F}$ | $\mathbf{H4F}$ | $\mathbf{H5F}$ | $\mathbf{H6F}$ | $\mathbf{H7F}$ |
| --- | --- | --- | --- | --- | --- | --- | --- |
| $H1F$ | 0.0000 | 0.0909 | 0.0909 | 0.0455 | 0.0455 | 0.0909 | 0.0455 |
| $H2F$ | 0.0909 | 0.0000 | 0.0455 | 0.0909 | 0.0909 | 0.0455 | 0.0909 |
| $H3F$ | 0.1364 | 0.0455 | 0.0000 | 0.0455 | 0.0909 | 0.0455 | 0.0909 |
| $H4F$ | 0.1364 | 0.1818 | 0.1364 | 0.0000 | 0.1818 | 0.1364 | 0.0909 |
| $H5F$ | 0.0455 | 0.0909 | 0.0455 | 0.0909 | 0.0000 | 0.0455 | 0.0455 |
| $H6F$ | 0.0909 | 0.0909 | 0.0909 | 0.0909 | 0.0455 | 0.0000 | 0.0909 |
| $H7F$ | 0.1364 | 0.1818 | 0.1818 | 0.1364 | 0.1818 | 0.1818 | 0.0000 |

Table SA3. The normalized direct-matrix of expert 3

| $\boldsymbol{X}$ | $\mathbf{H1F}$ | $\mathbf{H2F}$ | $\mathbf{H3F}$ | $\mathbf{H4F}$ | $\mathbf{H5F}$ | $\mathbf{H6F}$ | $\mathbf{H7F}$ |
| --- | --- | --- | --- | --- | --- | --- | --- |
| $H1F$ | 0.0000 | 0.0909 | 0.0455 | 0.0909 | 0.0455 | 0.0455 | 0.0909 |
| $H2F$ | 0.0909 | 0.0000 | 0.0455 | 0.0455 | 0.0455 | 0.0909 | 0.0455 |
| $H3F$ | 0.1364 | 0.1818 | 0.0000 | 0.1364 | 0.1364 | 0.1818 | 0.1818 |
| $H4F$ | 0.1364 | 0.1818 | 0.1818 | 0.0000 | 0.1818 | 0.1818 | 0.1364 |
| $H5F$ | 0.0455 | 0.0909 | 0.0909 | 0.0909 | 0.0000 | 0.0909 | 0.0909 |
| $H6F$ | 0.1818 | 0.0455 | 0.0455 | 0.0455 | 0.0455 | 0.0000 | 0.0455 |
| $H7F$ | 0.0909 | 0.0455 | 0.0909 | 0.0455 | 0.0909 | 0.0455 | 0.0000 |

Table SA4. The normalized direct-matrix of expert 4

| $\boldsymbol{X}$ | $\mathbf{H1F}$ | $\mathbf{H2F}$ | $\mathbf{H3F}$ | $\mathbf{H4F}$ | $\mathbf{H5F}$ | $\mathbf{H6F}$ | $\mathbf{H7F}$ |
| --- | --- | --- | --- | --- | --- | --- | --- |
| $H1F$ | 0.0000 | 0.1364 | 0.1364 | 0.1364 | 0.1364 | 0.1364 | 0.1364 |
| $H2F$ | 0.1364 | 0.0000 | 0.1364 | 0.1364 | 0.1364 | 0.1818 | 0.1364 |
| $H3F$ | 0.0909 | 0.0455 | 0.0000 | 0.0909 | 0.0455 | 0.0909 | 0.0909 |
| $H4F$ | 0.0455 | 0.0909 | 0.0909 | 0.0000 | 0.0909 | 0.1364 | 0.0909 |
| $H5F$ | 0.1818 | 0.1364 | 0.1364 | 0.0909 | 0.0000 | 0.1364 | 0.1364 |
| $H6F$ | 0.0909 | 0.1364 | 0.1364 | 0.1364 | 0.1364 | 0.0000 | 0.1818 |
| $H7F$ | 0.1364 | 0.1818 | 0.1818 | 0.1818 | 0.1364 | 0.1818 | 0.0000 |

Table SA5. The normalized direct-matrix of expert 5

| $\boldsymbol{X}$ | $\mathbf{H1F}$ | $\mathbf{H2F}$ | $\mathbf{H3F}$ | $\mathbf{H4F}$ | $\mathbf{H5F}$ | $\mathbf{H6F}$ | $\mathbf{H7F}$ |
| --- | --- | --- | --- | --- | --- | --- | --- |
| $H1F$ | 0.0000 | 0.1304 | 0.1739 | 0.1739 | 0.1739 | 0.1304 | 0.1739 |
| $H2F$ | 0.1304 | 0.0000 | 0.1739 | 0.1739 | 0.1739 | 0.1739 | 0.1739 |
| $H3F$ | 0.0870 | 0.0870 | 0.0000 | 0.0870 | 0.0870 | 0.1304 | 0.0870 |
| $H4F$ | 0.0870 | 0.0870 | 0.0870 | 0.0000 | 0.0870 | 0.0870 | 0.0870 |
| $H5F$ | 0.1739 | 0.1304 | 0.1304 | 0.1304 | 0.0000 | 0.1304 | 0.1304 |
| $H6F$ | 0.1304 | 0.1304 | 0.1304 | 0.1304 | 0.1304 | 0.0000 | 0.1304 |
| $H7F$ | 0.0870 | 0.0870 | 0.0870 | 0.0435 | 0.0870 | 0.0870 | 0.0000 |

Table SA6. The normalized direct-matrix of expert 6

| $\boldsymbol{X}$ | $\mathbf{H1F}$ | $\mathbf{H2F}$ | $\mathbf{H3F}$ | $\mathbf{H4F}$ | $\mathbf{H5F}$ | $\mathbf{H6F}$ | $\mathbf{H7F}$ |
| --- | --- | --- | --- | --- | --- | --- | --- |
| $H1F$ | 0.0000 | 0.0556 | 0.0556 | 0.1111 | 0.0556 | 0.1111 | 0.0556 |
| $H2F$ | 0.0556 | 0.0000 | 0.1111 | 0.0556 | 0.1111 | 0.0556 | 0.0556 |
| $H3F$ | 0.1667 | 0.1667 | 0.0000 | 0.1667 | 0.1667 | 0.1667 | 0.1667 |
| $H4F$ | 0.1111 | 0.1111 | 0.1111 | 0.0000 | 0.1111 | 0.1111 | 0.1111 |
| $H5F$ | 0.0556 | 0.0556 | 0.0556 | 0.0556 | 0.0000 | 0.0556 | 0.0556 |
| $H6F$ | 0.0556 | 0.0556 | 0.0556 | 0.0556 | 0.0556 | 0.0000 | 0.0556 |
| $H7F$ | 0.1667 | 0.1667 | 0.1667 | 0.1667 | 0.1667 | 0.1667 | 0.0000 |

Table SA7. The normalized direct-matrix of expert 7

| $\boldsymbol{X}$ | $\mathbf{H1F}$ | $\mathbf{H2F}$ | $\mathbf{H3F}$ | $\mathbf{H4F}$ | $\mathbf{H5F}$ | $\mathbf{H6F}$ | $\mathbf{H7F}$ |
| --- | --- | --- | --- | --- | --- | --- | --- |
| $H1F$ | 0.0000 | 0.1667 | 0.1667 | 0.1667 | 0.1667 | 0.1667 | 0.1667 |
| $H2F$ | 0.1250 | 0.0000 | 0.1250 | 0.1667 | 0.1667 | 0.1667 | 0.1667 |
| $H3F$ | 0.0833 | 0.0417 | 0.0000 | 0.0417 | 0.0833 | 0.0417 | 0.0833 |
| $H4F$ | 0.1250 | 0.1250 | 0.1250 | 0.0000 | 0.1250 | 0.0833 | 0.1250 |
| $H5F$ | 0.1250 | 0.1667 | 0.1667 | 0.1667 | 0.0000 | 0.1667 | 0.1667 |
| $H6F$ | 0.1250 | 0.1667 | 0.1250 | 0.1667 | 0.1667 | 0.0000 | 0.1667 |
| $H7F$ | 0.0833 | 0.0417 | 0.0833 | 0.0833 | 0.0417 | 0.0417 | 0.0000 |

Table SA8. The normalized direct-matrix of expert 8

| $\boldsymbol{X}$ | $\mathbf{H1F}$ | $\mathbf{H2F}$ | $\mathbf{H3F}$ | $\mathbf{H4F}$ | $\mathbf{H5F}$ | $\mathbf{H6F}$ | $\mathbf{H7F}$ |
| --- | --- | --- | --- | --- | --- | --- | --- |
| $H1F$ | 0.0000 | 0.1000 | 0.1000 | 0.1000 | 0.1000 | 0.0500 | 0.1000 |
| $H2F$ | 0.0500 | 0.0000 | 0.1000 | 0.1000 | 0.1000 | 0.1500 | 0.1000 |
| $H3F$ | 0.1000 | 0.1000 | 0.0000 | 0.2000 | 0.1500 | 0.2000 | 0.1500 |
| $H4F$ | 0.0500 | 0.1000 | 0.0500 | 0.0000 | 0.1000 | 0.1000 | 0.1000 |
| $H5F$ | 0.1000 | 0.2000 | 0.2000 | 0.2000 | 0.0000 | 0.1500 | 0.1500 |
| $H6F$ | 0.0500 | 0.1000 | 0.0500 | 0.0500 | 0.0500 | 0.0000 | 0.1000 |
| $H7F$ | 0.1500 | 0.1500 | 0.1500 | 0.1500 | 0.2000 | 0.1500 | 0.0000 |

Table SA9. The normalized direct-matrix of expert 9

| $\boldsymbol{X}$ | $\mathbf{H1F}$ | $\mathbf{H2F}$ | $\mathbf{H3F}$ | $\mathbf{H4F}$ | $\mathbf{H5F}$ | $\mathbf{H6F}$ | $\mathbf{H7F}$ |
| --- | --- | --- | --- | --- | --- | --- | --- |
| $H1F$ | 0.0000 | 0.0476 | 0.0952 | 0.0476 | 0.0952 | 0.0476 | 0.0476 |
| $H2F$ | 0.1429 | 0.0000 | 0.1905 | 0.1429 | 0.1905 | 0.1429 | 0.1905 |
| $H3F$ | 0.1905 | 0.1429 | 0.0000 | 0.1905 | 0.1429 | 0.1905 | 0.1429 |
| $H4F$ | 0.0476 | 0.0476 | 0.0476 | 0.0000 | 0.0476 | 0.0476 | 0.0476 |
| $H5F$ | 0.1429 | 0.1429 | 0.1429 | 0.1429 | 0.0000 | 0.1429 | 0.1429 |
| $H6F$ | 0.0952 | 0.0952 | 0.0952 | 0.0952 | 0.0952 | 0.0000 | 0.0952 |
| $H7F$ | 0.0476 | 0.0476 | 0.0476 | 0.0476 | 0.0476 | 0.0476 | 0.0000 |

Table SA10. The normalized direct-matrix of expert 10

| $\boldsymbol{X}$ | $\mathbf{H1F}$ | $\mathbf{H2F}$ | $\mathbf{H3F}$ | $\mathbf{H4F}$ | $\mathbf{H5F}$ | $\mathbf{H6F}$ | $\mathbf{H7F}$ |
| --- | --- | --- | --- | --- | --- | --- | --- |
| $H1F$ | 0.0000 | 0.1739 | 0.1304 | 0.1739 | 0.1739 | 0.1739 | 0.1739 |
| $H2F$ | 0.0870 | 0.0000 | 0.0870 | 0.0435 | 0.0870 | 0.0870 | 0.1304 |
| $H3F$ | 0.0435 | 0.0870 | 0.0000 | 0.0870 | 0.0435 | 0.0870 | 0.1739 |
| $H4F$ | 0.1304 | 0.1304 | 0.1304 | 0.0000 | 0.1304 | 0.1304 | 0.1304 |
| $H5F$ | 0.0435 | 0.0435 | 0.0870 | 0.0435 | 0.0000 | 0.0870 | 0.0435 |
| $H6F$ | 0.1304 | 0.1304 | 0.1739 | 0.1304 | 0.1304 | 0.0000 | 0.1739 |
| $H7F$ | 0.1739 | 0.1304 | 0.1304 | 0.1304 | 0.1739 | 0.1304 | 0.0000 |

able SA11. The normalized direct-matrix of expert 11

| $\boldsymbol{X}$ | $\mathbf{H1F}$ | $\mathbf{H2F}$ | $\mathbf{H3F}$ | $\mathbf{H4F}$ | $\mathbf{H5F}$ | $\mathbf{H6F}$ | $\mathbf{H7F}$ |
| --- | --- | --- | --- | --- | --- | --- | --- |
| $H1F$ | 0.0000 | 0.1818 | 0.1818 | 0.1364 | 0.1818 | 0.1364 | 0.1818 |
| $H2F$ | 0.0909 | 0.0000 | 0.1364 | 0.1364 | 0.1364 | 0.1364 | 0.1364 |
| $H3F$ | 0.0909 | 0.0455 | 0.0000 | 0.0909 | 0.0455 | 0.0455 | 0.0909 |
| $H4F$ | 0.0455 | 0.0455 | 0.0909 | 0.0000 | 0.1364 | 0.0909 | 0.1364 |
| $H5F$ | 0.0909 | 0.0455 | 0.0455 | 0.0455 | 0.0000 | 0.0000 | 0.0455 |
| $H6F$ | 0.0455 | 0.0455 | 0.0909 | 0.0909 | 0.0909 | 0.0000 | 0.0455 |
| $H7F$ | 0.1364 | 0.0455 | 0.0909 | 0.0455 | 0.0000 | 0.0000 | 0.0000 |

Table SA12. The normalized direct-matrix of expert 12

| $\boldsymbol{X}$ | $\mathbf{H1F}$ | $\mathbf{H2F}$ | $\mathbf{H3F}$ | $\mathbf{H4F}$ | $\mathbf{H5F}$ | $\mathbf{H6F}$ | $\mathbf{H7F}$ |
| --- | --- | --- | --- | --- | --- | --- | --- |
| $H1F$ | 0.0000 | 0.0417 | 0.0417 | 0.0417 | 0.0417 | 0.0833 | 0.0417 |
| $H2F$ | 0.0833 | 0.0000 | 0.0833 | 0.0833 | 0.0833 | 0.0833 | 0.0833 |
| $H3F$ | 0.1667 | 0.1250 | 0.0000 | 0.1667 | 0.1250 | 0.1250 | 0.1667 |
| $H4F$ | 0.1250 | 0.1250 | 0.1250 | 0.0000 | 0.1667 | 0.1250 | 0.1250 |
| $H5F$ | 0.1667 | 0.1667 | 0.1667 | 0.1667 | 0.0000 | 0.1667 | 0.1667 |
| $H6F$ | 0.1667 | 0.1667 | 0.1250 | 0.1250 | 0.1250 | 0.0000 | 0.1250 |
| $H7F$ | 0.1250 | 0.1250 | 0.1250 | 0.0833 | 0.1250 | 0.1667 | 0.0000 |

**Supporting information section B**

Table SB1. The total-relation matrix of expert 1

| $\boldsymbol{T}$ | $\mathbf{H1F}$ | $\mathbf{H2F}$ | $\mathbf{H3F}$ | $\mathbf{H4F}$ | $\mathbf{H5F}$ | $\mathbf{H6F}$ | $\mathbf{H7F}$ |
| --- | --- | --- | --- | --- | --- | --- | --- |
| $H1F$ | 0.3912 | 0.6418 | 0.5368 | 0.6674 | 0.5625 | 0.6643 | 0.6964 |
| $H2F$ | 0.4951 | 0.4661 | 0.5186 | 0.6459 | 0.5772 | 0.6092 | 0.6740 |
| $H3F$ | 0.4370 | 0.5911 | 0.3744 | 0.5794 | 0.5181 | 0.6118 | 0.6046 |
| $H4F$ | 0.2578 | 0.3459 | 0.3083 | 0.2763 | 0.3221 | 0.3588 | 0.3753 |
| $H5F$ | 0.5470 | 0.6089 | 0.5376 | 0.6682 | 0.4419 | 0.6665 | 0.6973 |
| $H6F$ | 0.4546 | 0.5366 | 0.4761 | 0.5580 | 0.4976 | 0.4365 | 0.5822 |
| $H7F$ | 0.2060 | 0.2815 | 0.2157 | 0.2545 | 0.2270 | 0.2906 | 0.2221 |

Table SB2. The total-relation matrix of expert 2

| $\boldsymbol{T}$ | $\mathbf{H1F}$ | $\mathbf{H2F}$ | $\mathbf{H3F}$ | $\mathbf{H4F}$ | $\mathbf{H5F}$ | $\mathbf{H6F}$ | $\mathbf{H7F}$ |
| --- | --- | --- | --- | --- | --- | --- | --- |
| $H1F$ | 0.0882 | 0.1726 | 0.1622 | 0.1133 | 0.1276 | 0.1554 | 0.1101 |
| $H2F$ | 0.1849 | 0.1113 | 0.1394 | 0.1664 | 0.1871 | 0.1336 | 0.1579 |
| $H3F$ | 0.2196 | 0.1476 | 0.0910 | 0.1219 | 0.1785 | 0.1289 | 0.1535 |
| $H4F$ | 0.2909 | 0.3379 | 0.2742 | 0.1387 | 0.3262 | 0.2628 | 0.2111 |
| $H5F$ | 0.1262 | 0.1721 | 0.1185 | 0.1500 | 0.0838 | 0.1136 | 0.1054 |
| $H6F$ | 0.1970 | 0.2020 | 0.1877 | 0.1725 | 0.1559 | 0.0966 | 0.1668 |
| $H7F$ | 0.3204 | 0.3665 | 0.3389 | 0.2818 | 0.3538 | 0.3248 | 0.1499 |

Table SB3. The total-relation matrix of expert 3

| $\boldsymbol{T}$ | $\mathbf{H1F}$ | $\mathbf{H2F}$ | $\mathbf{H3F}$ | $\mathbf{H4F}$ | $\mathbf{H5F}$ | $\mathbf{H6F}$ | $\mathbf{H7F}$ |
| --- | --- | --- | --- | --- | --- | --- | --- |
| $H1F$ | 0.0965 | 0.1724 | 0.1168 | 0.1481 | 0.1216 | 0.1323 | 0.1660 |
| $H2F$ | 0.1687 | 0.0739 | 0.1017 | 0.0999 | 0.1059 | 0.1567 | 0.1130 |
| $H3F$ | 0.3277 | 0.3414 | 0.1483 | 0.2578 | 0.2794 | 0.3420 | 0.3302 |
| $H4F$ | 0.3402 | 0.3574 | 0.3149 | 0.1502 | 0.3280 | 0.3581 | 0.3074 |
| $H5F$ | 0.1652 | 0.1928 | 0.1713 | 0.1640 | 0.0951 | 0.1940 | 0.1857 |
| $H6F$ | 0.2530 | 0.1268 | 0.1072 | 0.1081 | 0.1116 | 0.0779 | 0.1221 |
| $H7F$ | 0.1791 | 0.1351 | 0.1544 | 0.1135 | 0.1608 | 0.1331 | 0.0866 |

Table SB4. The total-relation matrix of expert 4

| $\boldsymbol{T}$ | $\mathbf{H1F}$ | $\mathbf{H2F}$ | $\mathbf{H3F}$ | $\mathbf{H4F}$ | $\mathbf{H5F}$ | $\mathbf{H6F}$ | $\mathbf{H7F}$ |
| --- | --- | --- | --- | --- | --- | --- | --- |
| $H1F$ | 0.3356 | 0.4780 | 0.5196 | 0.5005 | 0.4580 | 0.5379 | 0.5003 |
| $H2F$ | 0.4725 | 0.3773 | 0.5405 | 0.5207 | 0.4764 | 0.5948 | 0.5218 |
| $H3F$ | 0.2759 | 0.2544 | 0.2331 | 0.3063 | 0.2422 | 0.3262 | 0.3046 |
| $H4F$ | 0.2783 | 0.3321 | 0.3610 | 0.2644 | 0.3182 | 0.4104 | 0.3490 |
| $H5F$ | 0.4979 | 0.4838 | 0.5259 | 0.4699 | 0.3436 | 0.5430 | 0.5064 |
| $H6F$ | 0.4227 | 0.4819 | 0.5238 | 0.5047 | 0.4604 | 0.4223 | 0.5382 |
| $H7F$ | 0.5135 | 0.5758 | 0.6259 | 0.6043 | 0.5179 | 0.6481 | 0.4488 |

Table SB5. The total-relation matrix of expert 5

| $\boldsymbol{T}$ | $\mathbf{H1F}$ | $\mathbf{H2F}$ | $\mathbf{H3F}$ | $\mathbf{H4F}$ | $\mathbf{H5F}$ | $\mathbf{H6F}$ | $\mathbf{H7F}$ |
| --- | --- | --- | --- | --- | --- | --- | --- |
| $H1F$ | 0.3444 | 0.4403 | 0.5292 | 0.5081 | 0.5089 | 0.4775 | 0.5292 |
| $H2F$ | 0.4756 | 0.3400 | 0.5462 | 0.5243 | 0.5252 | 0.5279 | 0.5462 |
| $H3F$ | 0.3001 | 0.2881 | 0.2432 | 0.3102 | 0.3107 | 0.3470 | 0.3232 |
| $H4F$ | 0.2837 | 0.2724 | 0.3055 | 0.2133 | 0.2938 | 0.2946 | 0.3055 |
| $H5F$ | 0.4609 | 0.4098 | 0.4610 | 0.4426 | 0.3279 | 0.4433 | 0.4610 |
| $H6F$ | 0.4092 | 0.3929 | 0.4407 | 0.4230 | 0.4237 | 0.3095 | 0.4407 |
| $H7F$ | 0.2723 | 0.2615 | 0.2933 | 0.2448 | 0.2820 | 0.2828 | 0.2133 |

Table SB6. The total-relation matrix of expert 6

| $\boldsymbol{T}$ | $\mathbf{H1F}$ | $\mathbf{H2F}$ | $\mathbf{H3F}$ | $\mathbf{H4F}$ | $\mathbf{H5F}$ | $\mathbf{H6F}$ | $\mathbf{H7F}$ |
| --- | --- | --- | --- | --- | --- | --- | --- |
| $H1F$ | 0.0965 | 0.1491 | 0.1420 | 0.1965 | 0.1570 | 0.2068 | 0.1349 |
| $H2F$ | 0.1547 | 0.1020 | 0.1924 | 0.1547 | 0.2127 | 0.1628 | 0.1399 |
| $H3F$ | 0.3511 | 0.3511 | 0.1916 | 0.3511 | 0.3696 | 0.3696 | 0.3177 |
| $H4F$ | 0.2458 | 0.2458 | 0.2341 | 0.1458 | 0.2587 | 0.2587 | 0.2224 |
| $H5F$ | 0.1294 | 0.1294 | 0.1232 | 0.1294 | 0.0835 | 0.1362 | 0.1170 |
| $H6F$ | 0.1294 | 0.1294 | 0.1232 | 0.1294 | 0.1362 | 0.0835 | 0.1170 |
| $H7F$ | 0.3511 | 0.3511 | 0.3344 | 0.3511 | 0.3696 | 0.3696 | 0.1748 |

Table SB7. The total-relation matrix of expert 7

| $\boldsymbol{T}$ | $\mathbf{H1F}$ | $\mathbf{H2F}$ | $\mathbf{H3F}$ | $\mathbf{H4F}$ | $\mathbf{H5F}$ | $\mathbf{H6F}$ | $\mathbf{H7F}$ |
| --- | --- | --- | --- | --- | --- | --- | --- |
| $H1F$ | 0.3617 | 0.5184 | 0.5641 | 0.5599 | 0.5385 | 0.4984 | 0.6032 |
| $H2F$ | 0.4479 | 0.3501 | 0.5021 | 0.5324 | 0.5109 | 0.4740 | 0.5722 |
| $H3F$ | 0.2228 | 0.1934 | 0.1720 | 0.2104 | 0.2353 | 0.1859 | 0.2635 |
| $H4F$ | 0.3758 | 0.3849 | 0.4201 | 0.3047 | 0.3999 | 0.3383 | 0.4479 |
| $H5F$ | 0.4559 | 0.4999 | 0.5439 | 0.5399 | 0.3764 | 0.4806 | 0.5816 |
| $H6F$ | 0.4479 | 0.4930 | 0.5021 | 0.5324 | 0.5109 | 0.3311 | 0.5722 |
| $H7F$ | 0.2197 | 0.1890 | 0.2442 | 0.2398 | 0.1977 | 0.1805 | 0.1815 |

Table SB8. The total-relation matrix of expert 8

| $\boldsymbol{T}$ | $\mathbf{H1F}$ | $\mathbf{H2F}$ | $\mathbf{H3F}$ | $\mathbf{H4F}$ | $\mathbf{H5F}$ | $\mathbf{H6F}$ | $\mathbf{H7F}$ |
| --- | --- | --- | --- | --- | --- | --- | --- |
| $H1F$ | 0.1442 | 0.3019 | 0.2751 | 0.3144 | 0.2887 | 0.2755 | 0.2882 |
| $H2F$ | 0.1970 | 0.2195 | 0.2791 | 0.3190 | 0.2931 | 0.3689 | 0.2963 |
| $H3F$ | 0.3033 | 0.4058 | 0.2692 | 0.4981 | 0.4214 | 0.5066 | 0.4250 |
| $H4F$ | 0.1763 | 0.2808 | 0.2131 | 0.1960 | 0.2652 | 0.2929 | 0.2663 |
| $H5F$ | 0.3270 | 0.5188 | 0.4711 | 0.5385 | 0.3264 | 0.5101 | 0.4591 |
| $H6F$ | 0.1534 | 0.2445 | 0.1820 | 0.2080 | 0.1928 | 0.1655 | 0.2334 |
| $H7F$ | 0.3615 | 0.4716 | 0.4270 | 0.4880 | 0.4845 | 0.4934 | 0.3182 |

Table SB9. The total-relation matrix of expert 9

| $\boldsymbol{T}$ | $\mathbf{H1F}$ | $\mathbf{H2F}$ | $\mathbf{H3F}$ | $\mathbf{H4F}$ | $\mathbf{H5F}$ | $\mathbf{H6F}$ | $\mathbf{H7F}$ |
| --- | --- | --- | --- | --- | --- | --- | --- |
| $H1F$ | 0.1108 | 0.1354 | 0.1874 | 0.1563 | 0.1874 | 0.1495 | 0.1539 |
| $H2F$ | 0.3735 | 0.2010 | 0.3916 | 0.3735 | 0.3916 | 0.3572 | 0.4102 |
| $H3F$ | 0.3988 | 0.3148 | 0.2195 | 0.3988 | 0.3445 | 0.3815 | 0.3577 |
| $H4F$ | 0.1228 | 0.1077 | 0.1173 | 0.0773 | 0.1173 | 0.1174 | 0.1223 |
| $H5F$ | 0.3376 | 0.2961 | 0.3225 | 0.3376 | 0.1975 | 0.3230 | 0.3364 |
| $H6F$ | 0.2349 | 0.2060 | 0.2243 | 0.2349 | 0.2243 | 0.1377 | 0.2341 |
| $H7F$ | 0.1228 | 0.1077 | 0.1173 | 0.1228 | 0.1173 | 0.1174 | 0.0769 |

Table SB10. The total-relation matrix of expert 10

| $\boldsymbol{T}$ | $\mathbf{H1F}$ | $\mathbf{H2F}$ | $\mathbf{H3F}$ | $\mathbf{H4F}$ | $\mathbf{H5F}$ | $\mathbf{H6F}$ | $\mathbf{H7F}$ |
| --- | --- | --- | --- | --- | --- | --- | --- |
| $H1F$ | 0.2945 | 0.4754 | 0.4626 | 0.4389 | 0.4980 | 0.4763 | 0.5293 |
| $H2F$ | 0.2456 | 0.1831 | 0.2749 | 0.2075 | 0.2763 | 0.2636 | 0.3298 |
| $H3F$ | 0.2181 | 0.2687 | 0.2005 | 0.2480 | 0.2454 | 0.2678 | 0.3714 |
| $H4F$ | 0.3511 | 0.3780 | 0.3949 | 0.2336 | 0.3950 | 0.3787 | 0.4230 |
| $H5F$ | 0.1515 | 0.1645 | 0.2117 | 0.1519 | 0.1288 | 0.2016 | 0.1886 |
| $H6F$ | 0.3751 | 0.4039 | 0.4573 | 0.3728 | 0.4220 | 0.2892 | 0.4886 |
| $H7F$ | 0.4067 | 0.4027 | 0.4209 | 0.3717 | 0.4575 | 0.4048 | 0.3352 |

Table SB11. The total-relation matrix of expert 11

| $\boldsymbol{T}$ | $\mathbf{H1F}$ | $\mathbf{H2F}$ | $\mathbf{H3F}$ | $\mathbf{H4F}$ | $\mathbf{H5F}$ | $\mathbf{H6F}$ | $\mathbf{H7F}$ |
| --- | --- | --- | --- | --- | --- | --- | --- |
| $H1F$ | 0.1559 | 0.2793 | 0.3409 | 0.2785 | 0.3232 | 0.2365 | 0.3427 |
| $H2F$ | 0.2025 | 0.0927 | 0.2624 | 0.2430 | 0.2500 | 0.2106 | 0.2638 |
| $H3F$ | 0.1541 | 0.1023 | 0.0863 | 0.1560 | 0.1216 | 0.0985 | 0.1720 |
| $H4F$ | 0.1296 | 0.1063 | 0.1779 | 0.0802 | 0.2060 | 0.1384 | 0.2172 |
| $H5F$ | 0.1357 | 0.0892 | 0.1079 | 0.0977 | 0.0591 | 0.0444 | 0.1101 |
| $H6F$ | 0.1084 | 0.0941 | 0.1597 | 0.1501 | 0.1556 | 0.0485 | 0.1222 |
| $H7F$ | 0.1867 | 0.1019 | 0.1653 | 0.1123 | 0.0759 | 0.0571 | 0.0842 |

Table SB12. The total-relation matrix of expert 12

| $\boldsymbol{T}$ | $\mathbf{H1F}$ | $\mathbf{H2F}$ | $\mathbf{H3F}$ | $\mathbf{H4F}$ | $\mathbf{H5F}$ | $\mathbf{H6F}$ | $\mathbf{H7F}$ |
| --- | --- | --- | --- | --- | --- | --- | --- |
| $H1F$ | 0.1315 | 0.1593 | 0.1462 | 0.1460 | 0.1462 | 0.1937 | 0.1516 |
| $H2F$ | 0.2932 | 0.1953 | 0.2520 | 0.2517 | 0.2520 | 0.2726 | 0.2614 |
| $H3F$ | 0.4975 | 0.4289 | 0.2859 | 0.4282 | 0.3982 | 0.4319 | 0.4447 |
| $H4F$ | 0.4436 | 0.4119 | 0.3814 | 0.2697 | 0.4131 | 0.4124 | 0.3955 |
| $H5F$ | 0.5445 | 0.5055 | 0.4680 | 0.4674 | 0.3252 | 0.5062 | 0.4854 |
| $H6F$ | 0.4762 | 0.4433 | 0.3788 | 0.3783 | 0.3788 | 0.2999 | 0.3928 |
| $H7F$ | 0.4247 | 0.3943 | 0.3639 | 0.3305 | 0.3627 | 0.4266 | 0.2663 |
